# Inter-laboratory evolution of a model organism and its epistatic effects on mutagenesis screens

**DOI:** 10.1038/srep38001

**Published:** 2016-12-01

**Authors:** Michael D. Bradley, Devin Neu, Fatmagul Bahar, Roy D. Welch

**Affiliations:** 1 Department of Biology, Syracuse University, Syracuse NY 13244, USA.

## Abstract

In theory, a few naturally occurring evolutionary changes in the genome of a model organism may have little or no observable impact on its wild type phenotype, and yet still substantially impact the phenotypes of mutant strains through epistasis. To see if this is happening in a model organism, we obtained nine different laboratories’ wild type *Myxococcus xanthus* DK1622 “sublines” and sequenced each to determine if they had evolved after their physical separation. Under a common garden experiment, each subline satisfied the phenotypic prerequisites for wild type, but many differed to a significant degree in each of the four quantitative phenotypic traits we measured, with some sublines differing by several-fold. Genome resequencing identified 29 variants between the nine sublines, and eight had at least one unique variant within an Open Reading Frame (ORF). By disrupting the ORF MXAN7041 in two different sublines, we demonstrated substantial epistasis from these naturally occurring variants. The impact of such inter-laboratory wild type evolution is important to any genotype-to-phenotype study; an organism’s phenotype may be sensitive to small changes in genetic background, so that results from phenotypic screens and other related experiments might not agree with prior published results or the results from other laboratories.

Bacteria evolve quickly in the laboratory because they have short generation times and are typically grown in large numbers; this property has been exploited by researchers to study evolution in real-time under controlled experimental conditions. The most widely known experimental bacterial evolution project is the ongoing *Escherichia coli* long-term evolution experiment (LTEE), in which initially identical *E. coli* cultures were isolated and grown for tens of thousands of generations, during which they evolved different sets of mutations and phenotypic characteristics^1^,^2^,^3^,^4^. Other projects have addressed more specific topics in evolution, such as mutation rates^5^,^6^, fitness trajectories^7^, and the genetics of adaptation^8^,^9^,^10^. Most projects use relatively simple bacteria, such as *E. coli*, but a few use more complicated biofilm-forming bacteria, with larger genomes and multicellular phenotypes^11^,^12^.

These experimental bacterial evolution projects usually follow a similar protocol: they start with a founding strain, subject it to a controlled environment for many generations, and track changes to both genome and phenome through resequencing and phenotypic analysis. The ability to control the environment and track changes separates these projects from others that involve the collection, characterization, and sequencing of organisms isolated from their natural environment. In the laboratory, a researcher can control the type, duration, and intensity of selective pressure on the organism, and can calculate a reasonably accurate mutation rate. However, for an experimental model bacterium, the laboratory is its natural environment, and evolution continues during and between experiments.

If this evolution results in the laboratory’s wild type strain suddenly displaying an extreme change in phenotype, it would most likely be observed by the researcher, and the strain would be discarded and regrown from a frozen stock produced from an earlier generation. However, if this evolution results in little or no change in phenotype, it would most likely go unnoticed. Over time, these mutations would accumulate and, although the wild type strain’s phenotype would remain relatively unchanged, there is a real possibility they might change its phenotypic response to mutagenesis. This “gradual microevolution with epistasis” might also change the results from mutant screens, gene expression profiles, and the annotation of genes with respect to their biological function.

To determine the biological function of a gene thought to be involved in a complex phenotypic trait, a classical genetics approach is to alter or abolish it, and observe any change using phenotypic assays. If the change is deemed significant and specific to the trait, the biological function of the gene may be annotated as “important for” or “involved in” the manifestation of that trait. During the process of scaling-up from the annotation of a single gene to the annotation of all of the genes within the genome that are involved in producing that trait, the genome sequence of the wild type strain is assumed to be constant, usually represented by a reference sequence submitted to NCBI. Should the possibility of gradual microevolution with epistasis be incorporated into this long and iterative process? We attempted to find a real-world example.

*Myxococcus xanthus* is a gram-negative bacterium that displays a complex multicellular phenotype when several million cells are spotted as a dense swarm on an agar surface. If the agar is nutrient rich, the swarm will expand out from the point of inoculation through the coordination of two motility systems, adventurous (A motility) and social (S motility), in a process called swarming^13^. Alternatively, if the agar contains no nutrients, the starving swarm will appear to contract in a process called development, where cells first move to form aggregates of approximately 1×10^5^ cells each^14^, and then a subset of cells within each aggregate differentiate into dormant and environmentally resistant spores that germinate when nutrients become available. Explained in this way, the life cycle of *M. xanthus* is divided into two distinct halves, swarming and development, both of which can be described by measuring different phenotypic traits, such as the rate at which a swarm expands on nutrient agar, or the number of aggregates and spores that form during development. The phenotype of the wild type *M. xanthus* strain DK1622 has always been described as a range of assay results that measure traits like these.

*M. xanthus* DK1622 originated in the laboratory of Dale Kaiser at Stanford University^15^; it is one of two strains, the other being DZ2^16^, that have served as wild type for nearly four decades. Since its initial isolation in 1979^15^, DK1622 has been distributed to numerous research laboratories worldwide. To the best of our knowledge, each of the nine laboratory’s DK1622 strains, or “sublines” (designated S1-S9), used in this study has existed as an isolated and independent population since its distribution to that laboratory.

## Results

### Characterization of subline phenotypes in a common garden

Phenotype is considered the product of two variables, genotype and environment. To minimize environmental effects, all characterization experiments were performed under identical conditions in the same laboratory. All sublines were grown in aliquots from the same media preparations, each assay was performed on all sublines together using the same reagents and equipment, and images of each subline were acquired at the same time. These conditions defined our “common garden”.

**Figure 1:**
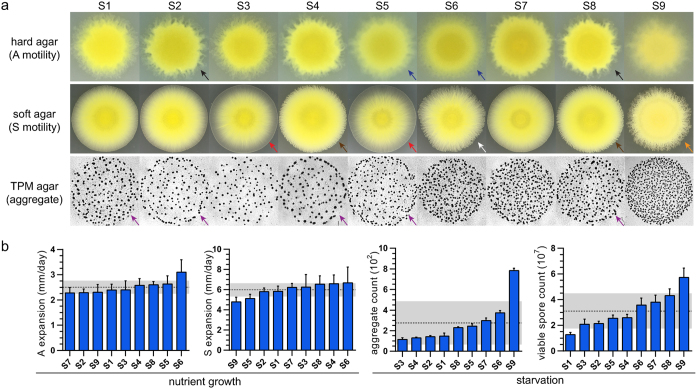
Characterization of DK1622 subline phenotypes in a common garden. The nine sublines were characterized for growth and development: (**a**) Qualitative comparisons of A motility (top), S motility (middle), and aggregation (bottom); Black arrows indicate pronounced edge flares; blue arrows indicate stunted edge flares; red arrows indicate a steep color gradient from the swarm center to the edge; brown arrows indicate directional edge flares; the white arrow indicates an irregular swarm shape; the orange arrow indicates long edge flares; purple arrows indicate a dense outer ring of aggregates. (**b**) Quantitative comparison of A & S motility, aggregation, and sporulation. The x-axes are ordered by increasing mean values. Error bars represent ±*SD* for each subline. The dashed line represents the population mean. The gray bar represents ±*SD* of the population mean.

A set of representative swarming images for each subline reveal clear differences in several qualitative aspects of phenotype (Fig. 1a); a side-by-side comparison of swarm expansion on hard agar (Fig. 1a, **top row)** reveals differences in swarm translucency and edge flare patterns. For example, sublines S5 and S6 are more translucent than S2 and S8, and the swarm edge flares are more pronounced in S2 and S8 than in S5 and S6. The same type of comparison on soft agar (Fig. 1a, **middle row)** reveals a range of swarm shapes, edge flare patterns, and color gradients extending from the center of the swarm to its edge. Sublines S3 and S5 are nearly circular in shape with a smooth swarm edge and a steep color gradient from dark yellow to translucent, while S4 and S8 are nearly circular in shape with a rough swarm edge consisting of numerous small and directional flares and a more subtle color gradient. Subline S6 is irregular in shape with a rough swarm edge consisting of a variety of flare shapes and an inconsistent color gradient, while S9 is circular in shape with long pronounced edge flares and no color gradient. A similar range of phenotypes is revealed by a side-by-side comparison of development on starvation agar (Fig. 1a, **bottom row)**. Subline aggregates range from small (S5) to large (S4), some of the sublines appear to have a greater distribution of individual aggregate sizes (S1), and some appear to have a dense ring of aggregates at the outermost edge (S1, S2, S4, S5, S8).

This qualitative characterization of sublines in a common garden reveals obvious differences, but their description is subjective, and so it is impossible to rank them using only this information. To achieve such a ranking, we selected four quantitative assays to represent the phenotype of DK1622 at both stages of its life cycle (Fig. 1b). Two swarming assays are used to measure changes in swarm diameter on hard and soft nutrient agar, and are considered estimates of the expansion rate for A and S motility systems. Two development assays are used to measure the number of aggregates and the number of spores that form on starvation agar, and are considered tests of self-organization and cellular differentiation. These four assays are commonly used by the research community to compare the phenotypes of mutant *M. xanthus* strains, using DK1622 as a wild type control. Because all of the strains in this study are DK1622 there can be no wild type control, and thus subline assay data are arranged by increasing means for each of these four traits.

The sublines differed significantly from one another in each of the four assays (Table 1), indicating that there are real and measurable differences between sublines. Because we observed a continuous distribution of means in each assay, we focused on the sublines at the phenotypic extremes, which we hereafter refer to as “outlier” sublines. We deemed a subline to be an outlier if its mean ± *SD* falls beyond one standard deviation of the total population mean for that assay. Based on this criterion, we identified two outliers with respect to development traits: S1 is an outlier for sporulation, and S9 is an outlier for both aggregation and sporulation. S9 is also an outlier with respect to S motility. To determine if the non-outlier sublines are different from each other, we repeated the analyses with S1 and S9 excluded, and the results remain significant for all traits except S motility (Supplementary Table S1).

### Subline variant screen

Each subline was sequenced and assembled using the original closed DK1622 genome sequence^17^ as a scaffold (NCBI accession number: NC_008095). An average of 8.1 million reads covering >99.4% of the scaffold genome were mapped for each subline. A total of 29 variants, consisting of 28 single nucleotide polymorphisms (SNPs) and one nucleotide deletion were identified among the nine sublines (Table 2). Any variant that occurred in two or more sublines was counted as one variant (i.e. the overlapping SNPs found in S2, S4, S5, and S8 were counted only once). Of the 28 SNPs, eight are transitions and 20 are transversions. Twenty-one variants (72%) are located within putative Open Reading Frames (ORFs), 11 of which are non-synonymous (i.e. they alter the protein coding sequence of their constituent ORF). Eight variants are found within noncoding regions. No evidence of chromosome structural variation was found in any of the sublines using the variant detection parameters described in Materials and Methods; a sampling of possible insertions and deletions with scores below the stated threshold were examined, and all were confirmed to be false positives by PCR (data not shown).

One part of the subline variant screen is in agreement with the common garden characterizations and functions as a useful control; sublines S2 and S4 are identical with respect to genotype, and they do not vary to a significant degree with respect to the four quantitative assays used in this study (*P* ≥ 0.818 for each trait). In addition, there are two results from these resequencing data that are notable, even though they are tangential to the primary focus of this study: First, Velicer *et al*. previously reported five variants in a derivative of the S3 subline in 2006^18^. These five variants were independently identified in this study along with three more, which may indicate that S3 has continued to accumulate mutations since 2006, or that the higher sequencing depth in this study was able to identify three variants that were not identified in the previous study. Second, two variants, a thymine-to-guanine transversion at position 830180 and a thymine-to-cytosine transition at position 7101832 are in all of the sublines. Because some of these sublines have been isolated from each other for more than 30 years, while the reference genome sequence was completed just over ten years ago, the simplest explanation for these two variants is that they represent sequencing errors within the reference genome.

### Targeted mutagenesis

S1 and S9 represent outlier sublines on different ends of the development rankings (Fig. 1b); S1 produces fewer spores than any other subline and is among the group of sublines (S1, S2, S3, and S4) that produce the fewest aggregates, whereas S9 produces more aggregates and more spores than any other subline. However, despite these differences, both sublines have functioned effectively as wild type controls for years in their respective laboratories.

The non-synonymous variants from either S1 or S9 are reasonable candidates for causing each subline’s outlier phenotype because they alter a protein’s sequence, and therefore may negatively affect its function. If it does, then disrupting the ORFs that harbor these variants in a more “average” non-outlier subline may shift its phenotype to resemble the phenotype of the corresponding outlier subline. To test this, we selected S8 to represent the average subline; S8 has a nearly average aggregate count, and its spore count is significantly different from both S1 (*P* < 0.001) and S9 (*P* = 0.023).

**Figure 2:**
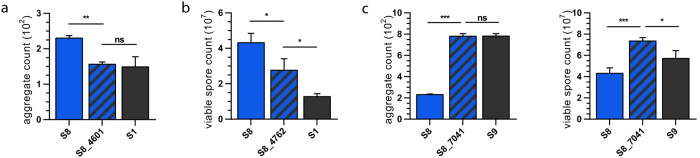
Characterization of S8 mutant strains. Insertion-disruption mutations constructed in S8 targeting ORFs MXAN4601, MXAN4762, and MXAN7041: The outlier subline is shown in gray; the parent subline is shown in blue; the mutant strain is shown as a cross of blue and gray. (**a**) Aggregate counts for S1, S8, and the mutant strain S8_4601. (**b**) Viable spore counts for S1, S8, and the mutant strain S8_4762. (**c**) Aggregate and viable spore counts for S9, S8, and the mutant strain S8_7041. Significance was determined using Tukey’s multiple comparison test: *P < 0.05; **P < 0.01; ***P < 0.001.

We constructed mutant strains containing single ORF insertion-disruptions in S8 for each of the ORFs harboring the three non-synonymous variants specific to the outliers S1 (located in MXAN4601 and MXAN4672) and S9 (located in MXAN7041); these new mutant strains are hereafter referred to as S8_4601, S8_4762, and S8_7041 respectively. Aggregation and sporulation assays were performed on each of the strains, and results were compared to both the parent subline (S8) and the corresponding outlier subline (either S1 or S9). For each mutant strain, the change in phenotype is reported as a percent change compared to the parent subline: S8_4601 exhibits a 33% reduction in aggregate count, which is significantly lower than S8 (*P* = 0.005) and matches its corresponding outlier subline, S1 (*P* = 0.863) (Fig. 2a). S8_4762 exhibits a 36% reduction in spore count, which is significantly lower than S8 (*P* = 0.021) and is intermediate between S8 and its corresponding outlier subline, S1 (Fig. 2b). Spore count for S8_4601 and aggregate count for S8_4762 did not differ from S8 to a significant degree (data not shown). S8_7041 exhibits a 238% increase in aggregate count, which is significantly higher than S8 (*P* < 0.001) and matches its corresponding outlier subline, S9 (*P* = 0.981) (Fig. 2c). S8_7041 also exhibits a 70% increase in spore count, which is significantly higher than both S8 (*P* = 0.001) and its corresponding outlier subline, S9 (*P* = 0.026) (Fig. 2c).

It is important to note that while the sporulation phenotypes of S8_4762 and S8_7041 do not exactly match that of their corresponding outlier sublines, they are both different from their parent subline in a way that moves their phenotypes closer to their corresponding outlier sublines. In other words, S8_4762 produces significantly fewer spores, which is more like S1, and S8_7041 produces significantly more spores, which is more like S9. These data support the idea that disrupting ORFs harboring these unique outlier variants in a more phenotypically average subline would shift its phenotype towards that of the outlier subline. These data also support the idea that the genetic variants in the outlier sublines are likely loss-of-function mutations, because if one of the variants were gain-of-function, then disrupting its corresponding ORF would likely have driven the phenotype of the average subline away from the phenotype of the outlier subline. In particular, the alanine-to-proline substitution in MXAN7041 of S9 almost certainly has a detrimental impact on its protein structure and function, due to severe conformational constraints imposed on it by proline.

**Figure 3:**
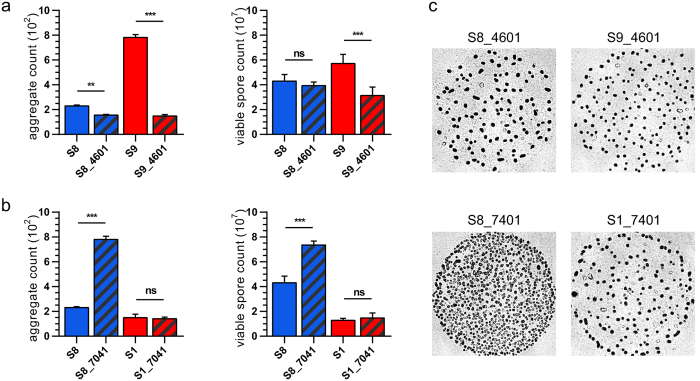
Epistasis in S1, S8, and S9 mutant strains. Insertion-disruption mutations constructed in S1, S8, and S9 targeting ORFS MXAN4601 and MXAN7041: The parent sublines are shown as blue or red; the mutant strain specific to each parent subline is shown as a cross of blue and gray or red and gray. (**a**) Aggregate and viable spore counts for MXAN4601 disruptions in S8 and S9. (**b**) Aggregate and viable spore counts for MXAN7041 disruptions in S8 and S1. (**c**) Representative aggregate images of mutant strains. Significance was determined using Tukey’s multiple comparison test: *P < 0.05; **P < 0.01; ***P < 0.001.

Results from the *M. xanthus* subline resequencing and analysis provide strong evidence for microevolution, and the purpose of our mutant analysis thus far has been to identify the candidate sublines and candidate ORFs most likely to provide strong evidence of epistasis. For the candidate sublines, we chose the average subline S8, together with the two phenotypically opposite outlier sublines S1 and S9. For the candidate ORFs we chose MXAN4601 and MXAN7041 because, at least for S8, their disruption significantly changes the results of the two most common development assays in opposite directions, so differences in their impact are easy to distinguish. To test for epistasis, one of the variant ORFs specific to each outlier subline, in this case MXAN4601, which is specific to S1, and MXAN7041, which is specific to S9, was disrupted in the opposing outlier subline. In other words, MXAN4601 was disrupted in S9 to create the mutant strain S9_4601, and MXAN7041 was disrupted in S1 to create the mutant strain S1_7041. The development phenotypes of both these strains were then compared to the development phenotypes of the same ORF disrupted in S8 (Fig. 3).

Differences in phenotype between a mutant strain and its parent subline are reported as a percent change compared to the parent subline: S9_4601 exhibits a relatively large reduction in aggregate count (81%) and spore count (45%) when compared to S8_4601, which exhibits a small reduction in aggregate count (33%) and no significant change in spore count (Fig. 3a). Most notably, S1_7041 exhibits no significant change in either aggregate or spore count, whereas S8_7041 exhibits a large increase in both (238% and 70%, respectively) (Fig. 3b). It is important to note that the same plasmid was used to disrupt MXAN7041 in sublines S1 and S8 (Supplementary Table S5); S1_7041 and S8_7041 have the same disruption genotype, and only differ by the naturally occurring variants listed in Table 1. Clearly, the variants between sublines S1 and S8 are having an epistatic effect on the disruption of MXAN7041, enough that this ORF would be annotated as “involved in development” in S8, but not S1.

## Discussion

Several previous studies have explored inter-laboratory microbial evolution. In 2007, Schacherer *et al*. identified nonrandom mutational events among several closely related laboratory sublines of *Saccharomyces cerevisiae*^19^. In 2008, Srivatsan *et al*. resequenced several *Bacillus subtilis* sublines and identified a previously unknown metabolism defect^20^. Finally, in 2010, Klockgether *et al*. identified discordant genotypes of the widely studied *Pseudomonas aeruginosa* strain PAO1^21^. To the best of our knowledge, this is the first study to examine the epistatic impact of microevolution on a microbial model organism, and to demonstrate that it was sufficient to change the initial annotation of a gene with respect to its biological function.

There are two mechanisms that could affect the evolution of *M. xanthus* in the laboratory. The first is genetic drift, which would have a stochastic effect on each subline’s genome and is almost certainly responsible for some of the genetic variation between sublines. The second is selection, which probably varies between laboratories and affects each subline differently. Selection may occur when cells are grown in liquid culture, favoring faster growing cells. It may occur when cells are grown as swarms on nutrient agar plates because inoculants for liquid cultures are taken from the swarm edge, favoring highly motile cells that may be overrepresented there. It may occur when cell cultures are made into frozen stocks, favoring cells that are better able to resist lysis when frozen. Certainly, purifying selection is always occurring, so that any cells with mutations that have a deleterious impact on growth or survival are removed. The variants in the nine *M. xanthus* sublines most likely were produced through some combination of these factors: drift, purifying selection, and selective pressures that were slightly different in each of the laboratories.

Resequencing of the nine *M. xanthus* DK1622 sublines clearly demonstrated microevolution, given that DK1622 has a single origin. Rather than randomly searching for an example of epistasis from that point, we decided to hedge our bets. This is why we singled out the two outlier sublines from the common garden, why we selected the three candidate ORFs we deemed most likely to be responsible for the sublines’ outlier phenotypes, and why we finally settled on the two ORFs whose disruption caused the strongest opposite changes in phenotype. In this study, our goal was to identify a statistically significant and entirely unambiguous example of naturally occurring epistasis, and we believe that we identified at least one: S1_7041 versus S8_7041. Future studies that employ a broader mutagenesis approach will likely produce an “epistasis distribution”, which may provide insight into the role of epistasis in the annotation of the *M. xanthus* genome.

The practical impact of epistasis on determining biological function in *M. xanthus* is evident in these results. Depending on which laboratory constructed the initial disruption, the ORF MXAN7041 may or may not have been identified as important for development. Disrupting MXAN7041 in S8 causes a more than 200% increase in aggregation and an almost 100% increase in sporulation, whereas disrupting that same ORF in S1 results in no change to either aggregation or sporulation. If a screen were performed for aggregation and sporulation mutants, S8_7041 would be identified as a gain-of-function mutation, whereas S1_7041 would not. This initial characterization would then guide all further experiments, as well as the annotation of this ORF with respect to its biological function.

Epistasis is a fundamental and frequently observed evolutionary phenomenon; thousands of examples have been identified^22^,^23^, and yet our ability to predict when and how epistasis will manifest remains very poor, and shows no real sign of improving. Perhaps this is because established evolutionary principles, like epistasis, seem to contradict the current interaction-network-as-a-circuit functional genomics paradigm, and this has produced a form of cognitive dissonance. As a result, a concept like gradual microevolution and epistasis can seem both obvious and confounding.

It is important to note that our findings are not from a controlled evolution project designed to demonstrate that gradual microevolution and epistasis could occur in isolates of a model bacterium when separated by time and distance. Rather, gradual microevolution and epistasis has occurred in *M. xanthus* wild type DK1622 laboratory stocks whose genomes were assumed to be identical and static. For the past ten years, the interpretation of mutant *M. xanthus* phenotype data has been based on the implicit assumption that the 2005 published reference sequence was the genome sequence for DK1622 in every laboratory that studied *M. xanthus*. This assumption is false, at least for the sublines in this study, all of which have at least one or two variants that are different from the reference. It seems very likely that this occurrence in *M. xanthus* is one example of a common phenomenon that is also happening in other model organisms.

## Materials and Methods

### Wild type strains

The first *M. xanthus* isolate moved into the laboratory was strain FB^24^. DK1622 is a derivative of FB that swarms on nutritive media and develops on starvation media^15^. In 2014, we received DK1622 from eight other laboratories that study *M. xanthus* as a model organism. Each subline was received on nutrient agar, grown in nutrient broth, concentrated, and preserved as a frozen stock. Our laboratory subline (S8) was cloned from the Kaiser strain archive at Stanford University in 2003. Supplementary Table S2 provides the source of each subline used in this study.

### Growth conditions

*M. xanthus* cells were cultured on CTTYE^25^ + 1.5% agar plates and incubated at 32 °C. Liquid cultures were prepared in agitating CTTYE liquid media. Media was supplemented with 40 μg/mL kanamycin sulfate for insertion-disruption mutants. Liquid cultures were harvested at a density of 5 × 10^8^ cells/mL and concentrated tenfold for characterization assays. Cells were washed with 5 mL TPM buffer^26^ before performing development assays.

### Strain characterization and analysis

DK1622 sublines were characterized as previously described^27^. Briefly, A and S expansion rates were measured by spotting sublines onto CTTYE + 1.5% (hard) and 0.4% (soft) agar plates. Growth rates were determined by dividing the swarm diameters by growth hours. Five temporally independent replicates were conducted. Aggregation assays were performed by spotting cells onto TPM + 1.5% agar. Images of resulting aggregates were captured after 24 hours using 20× bright-field microscopy (Nikon) and SPOT imaging software (SPOT Business Systems, LLC - USA). Resulting aggregates were manually counted. Sporulation assays were performed by spotting cells onto TPM + 1.5% agar, incubating for 120 hours, and scraping spore containing aggregates off the substrate. Cells were sonicated, diluted, and plated onto CTTYE. The resulting colonies are presumed to arise from a single germinated spore, and colony counts represent the number of viable spores (i.e. spores that survive heat and sonication). Three temporally independent replicates were conducted for both development assays. Statistical comparisons between subline phenotype data sets were made using a one-way ANOVA (α = 0.05), followed by *post hoc* analysis with Tukey’s multiple comparisons test with multiplicity adjusted *P* values. Aggregation data were log-transformed prior to analyses to achieve a normal distribution.

### Multiplex sequencing

Genomic DNA was extracted and purified using Zymo Universal Quick-DNA and DNA Clean & Concentrator miniprep kits (Zymo Research). Library preparation was performed using Nextera XT dual indexing kit (Illumina) according to the manufacturer’s instructions. Fragmented DNA libraries were verified with a 2100 Bioanalyzer (Agilent) and sequenced on a NextSeq500 pyrosequencer (Illumina) at the University of Pittsburgh Children’s Hospital Rangos Genomics Facility. Supplementary Table S3 provides a summary of read mappings for each sequenced subline.

### Genome assembly and variant detection

CLC Genomics Workbench (v8.0, Qiagen) was used to filter and assemble reads: Short reads (<60 base pairs), reads containing ambiguous nucleotides (“N”), low quality reads, duplicate reads (artificially inflates mapping coverage), and homopolymers were removed. Reads were assembled against the *M. xanthu*s DK1622 reference genome^17^ (NCBI accession number: NC_008095). Fixed variants were identified by restricting candidates to a frequency of ≥95% and a minimum sequencing depth of 15x.

### Mutant strain construction

Targeted insertion-disruption mutations were performed as previously described^27^. Briefly, fragments of target genes were ligated into a pCR 2.1 TOPO vector (Thermo Fisher) containing a kanamycin resistance selective marker, and replicated in TOP10 *E. coli* host cells (Invitrogen). Plasmids were integrated into the *M. xanthus* chromosome via homologous recombination^28^. Plasmid integration was confirmed by PCR. Supplementary Tables S4 and S5 provides a list of primer sequences and plasmids used in this study.

## Additional Information

**How to cite this article**: Bradley, M. D. *et al*. Inter-laboratory evolution of a model organism and its epistatic effects on mutagenesis screens. *Sci. Rep.* **6**, 38001; doi: 10.1038/srep38001 (2016).

**Publisher's note:** Springer Nature remains neutral with regard to jurisdictional claims in published maps and institutional affiliations.

## Supplementary Material

Supplementary Information

## Figures and Tables

**Table 1:** Summary of ANOVAs.

Quantitative trait	*F* (DFn, DFd)	*P* value
Expansion rate (A motility)	3.877 (8, 36)	0.0022
Expansion rate (S motility)	3.050 (8, 36)	0.01
Aggregate count	111.4 (8, 18)	<0.0001
Viable spore count	28.23 (8, 18)	<0.0001

A one-way analysis of variance (ANOVA) was performed for each quantitative trait. Degrees of freedom (DF) are calculated from the number of sublines (nine, numerator) and replicate experiments (three or five, denominator). Aggregate count data were normalized by log-transforming prior to analysis. Significant differences in subline means are indicated by *P* < 0.05.

**Table 2:** Subline variant screen.

Subline	Position	ORF	Nt change	AA change	TIGR annotation
S1	2678143	Nc	C → A		
	3849765	Nc	G → T		
	5766255	MXAN4601	G → C	G408R	non-ribosomal peptide synthase
	5964097	MXAN4762	C → A	G102C	6,7-dimethyl-8-ribityllumazine synthase
	6146569	MXAN4912	G → A	Syn	hypothetical protein
	8023083	MXAN6505	C → A	Syn	sulfate permease
	9009601	MXAN7384	T → A	Syn	conserved hypothetical protein
	9089098	MXAN7464	C → A	Syn	hypothetical protein
S2, S4	3591006	MXAN3061	T → C	V140A	α-L-glutamate ligases, RimK family
	5591111	MXAN4513	G → T	L837I	conserved hypothetical protein
S3	1717943	MXAN1458	G → T	Syn	THUMP domain/methyltransferase domain protein
	2304200	MXAN1970	T → Δ	L72Fs	transcriptional regulator, ArsR family
	4545964	MXAN3780	G →T	Syn	patatin-like phospholipase family protein
	5258697	MXAN4292	G → C	Q654E	polyketide synthase
	5391338	MXAN4388	G → A	T199M	D-lysine 5,6-aminomutase, α subunit
	5893144	Nc	A → C		
	6287570	Nc	C → A		
	8333625	MXAN6783	G → A	Syn	decarboxylase, group II
S5	2727430	Nc	C → G		
	3591006	MXAN3061	T → C	V140A	α-L-glutamate ligases, RimK family
	5591111	MXAN4513	G → T	L837I	conserved hypothetical protein
	6423875	MXAN5143	C → T	Syn	tol-pal system protein YbgF
	6465733	MXAN5175	T → G	B139A	hypothetical protein
	8966988	Nc	T → C		
S6	1523890	MXAN1297	C → A	R288S	putative serine/threonine protein kinase
	1985177	Nc	G → T		
S7	2310245	Nc	C → G		
S8	3591006	MXAN3061	T → C	V140A	α-L-glutamate ligases, RimK family
	4880522	MXAN4000	G → T	Syn	non-ribosomal peptide synthetase
	5180405	MXAN4219	G → A	Syn	α keto acid dehydrogenase complex
	5591111	MXAN4513	G → T	L837I	conserved hypothetical protein
S9	8617589	MXAN7041	C → G	A80P	cyclic nucleotide-binding domain protein
	8617592	MXAN7041	C → T	V79M	cyclic nucleotide-binding domain protein

Nt, nucleotide; Nc, noncoding; AA, amino acid; Δ, deletion; Syn, synonymous; Fs, frameshift.

Sublines S2 and S4 are listed together because they have identical variants.
