# Supplementary material for: Inter-laboratory evolution of a model organism and its epistatic effects on mutagenesis screens
Source: Sci Rep. 2016 Dec 1;6:38001. doi: 10.1038/srep38001 (PMC5131308; doi:10.1038/srep38001)
Supplement: Supplementary Information [file srep38001-s1.pdf]

Supplementary Information

Inter-laboratory evolution of a model organism and its epistatic effects on mutagenesis screens

Michael D. Bradley, Devin Neu, Fatmagul Bahar, and Roy D. Welch*

Inventory of Supplementary Information:

Table S1: Summary of ANOVAs with outliers removed.
Table S2: Source of *M. xanthus* sublines used in this study.
Table S3: Summary of read mappings.
Table S4: Primers used to generate DNA fragments for plasmids.
Table S5: Plasmids used to construct mutant strains.

Supplementary Table S1. Summary of ANOVAs with outliers removed.

Quantitative trait	*F* (DFn, DFd)	*P* value
Expansion rate (A motility)	4.340 (6, 28)	0.0032
Expansion rate (S motility)	1.776 (6, 28)	0.1405
Aggregate count	78.81 (6, 14)	< 0.0001
Viable spore count	13.34 (6, 14)	< 0.0001

A one-way analysis of variance (ANOVA) was performed for each quantitative trait with sublines S1 and S9 removed from the analysis. Degrees of freedom (DF) are calculated from the number of sublines (nine, numerator) and replicate experiments (three or five, denominator). Aggregate count data were normalized by log-transforming prior to analysis. Significant differences in subline means are indicated by $P < 0.05$.

Supplementary Table S2. Source of *M. xanthus* sublines used in this study.

Subline	Source
S1	University of Georgia
S2	Augustana College
S3	Michigan St. University
S4	Syracuse University
S5	University of Iowa
S6	Wayne State University
S7	University of California, Davis
S8	Syracuse University
S9	University of Wyoming

Supplementary Table S3. Summary of read mappings.

Library	Mapped read count	Average length (bp)
S1	6,897,065	126.88
S2	12,922,299	128.15
S3	6,872,636	128.98
S4	6,199,545	126.16
S5	7,266,263	129.7
S6	6,433,845	126.76
S7	8,677,798	126.24
S8	9,871,644	126.64
S9	8,199,433	127.55

Supplementary Table S4. Primers used to generate DNA fragments for plasmids.

Target ORF	Primer Sequence	Plasmid
MXAN4601	F: CGAATACAACGTGGACCTCT	pS1.a
	R: AGGGAGTTGAACTGGAGGAT	
	C: GGAGAGCAAAGACTTTACCG	
MXAN4762	F: TTCGACGGTGACTTTCTACC	pS1.b
	R: GCCAAAAGTCACCGTCTTG	
	C: GACCAGATAGGGAAGTACGTG	
MXAN7041	F: ATCCTCTCGCAGGTGAGC	pS9.a
	R: ATTGAAGAGCGGGTTGCT	
	C: GACATGAAGGACCTCTACCAAC	

ORF, open reading frame; F, forward; R, reverse; C, confirmation.

Supplementary Table S5. Plasmids used to construct mutant strains.

Mutant strain	Parent subline	Plasmid	Source
S1_7041	S1	pS9.a	This study
S8_4601	S8	pS1.a	This study
S8_4762	S8	pS1.b	This study
S8_7041	S8	pS9.a	This study
S9_4601	S9	pS1.a	This study
